# Explicit B-spline regularization in diffeomorphic image registration

**DOI:** 10.3389/fninf.2013.00039

**Published:** 2013-12-23

**Authors:** Nicholas J. Tustison, Brian B. Avants

**Affiliations:** ^1^Department of Radiology and Medical Imaging, University of VirginiaCharlottesville, VA, USA; ^2^Penn Image Computing and Science Laboratory, Department of Radiology, University of PennsylvaniaPhiladelphia, PA, USA

**Keywords:** Advanced normalization tools, diffeomorphisms, directly manipulated free-form deformation, Insight Toolkit, spatial normalization

## Abstract

Diffeomorphic mappings are central to image registration due largely to their topological properties and success in providing biologically plausible solutions to deformation and morphological estimation problems. Popular diffeomorphic image registration algorithms include those characterized by time-varying and constant velocity fields, and symmetrical considerations. Prior information in the form of regularization is used to enforce transform plausibility taking the form of physics-based constraints or through some approximation thereof, e.g., Gaussian smoothing of the vector fields [a la Thirion's Demons (Thirion, 1998)]. In the context of the original Demons' framework, the so-called *directly manipulated free-form deformation (DMFFD)* (Tustison et al., 2009) can be viewed as a smoothing alternative in which explicit regularization is achieved through fast B-spline approximation. This characterization can be used to provide B-spline “flavored” diffeomorphic image registration solutions with several advantages. Implementation is open source and available through the Insight Toolkit and our Advanced Normalization Tools (ANTs) repository. A thorough comparative evaluation with the well-known SyN algorithm (Avants et al., 2008), implemented within the same framework, and its B-spline analog is performed using open labeled brain data and open source evaluation tools.

## 1. Introduction

Establishment of anatomical and functional correspondence is a crucial step toward gaining insight into biological processes. Neuroscience research efforts, such as characterizing brain morphology, require accurate and robust methods for producing such mappings. The extensive literature detailing methodology is evidence of the rich history of algorithmic development which continues contemporaneously. We highlight several key historical contributions which are particularly relevant to the work presented.

Free-form deformation (FFD) image registration, characterized by regularization based on the B-spline basis functions, has several advantages including algorithmic simplicity, good performance, and guaranteed parametric continuity. Current research was preceded by related work for geometric modeling (Sederberg and Parry, 1986) and originated with such important contributions as Szeliski and Coughlan (1997); Thévenaz et al. (1998), and Rueckert et al. (1999). Continued development within this early spline-based paradigm produced additional innovations such as integrated similarity metrics (e.g., Mattes et al., 2003), additional transformation constraints (e.g., Rohlfing et al., 2003), and notable open source implementations (e.g., Ibanez et al., 2005; Klein et al., 2010b; Modat et al., 2010; Shackleford et al., 2010).

Parallel to this branch of algorithmic progress are the informally denoted “dense transforms” perhaps best exemplified by Thirion's seminal contribution (Thirion, 1998). Relationships with earlier elastic (Bajcsy and Kovacic, 1989; Gee et al., 1993) and fluid (Christensen et al., 1996) registration methods are detailed in the works of Bro-Nielsen and Gramkow (1996) and Pennec et al. (1999) who observe that smoothing via Gaussian convolution, a defining characteristic of Demons, of the update or total displacement field is a greedy approximation for solving the partial differential equations governing the physics of an elastic or fluid deformation, respectively. However, the use of such approximations entails that physical properties, such as topological regularity, are no longer guaranteed.

It is interesting to note that within this context, traditional FFD algorithms can be viewed as a type of fluid-like Demons approach where, rather than projecting the update field to the space of regularized fields using Gaussian convolution, gradient fields are projected to a smooth space characterized by the B-spline basis functions. This analogy was hinted at in our earlier work (Tustison et al., 2009) where we showed that fitting the update field to a B-spline object using a fast approximation routine (Tustison and Gee, 2006) is equivalent to a preconditioning of the standard gradient used in gradient descent-based FFD optimization. This preconditioning is used to mitigate the hemstitching effect induced by the ill-conditioned nature of the traditional gradient-based FFD formulation^1^. We denoted this new FFD variant as *directly manipulated free-form deformation* (DMFFD) and, as part of the ITKv4 refactoring efforts, has been implemented for use with the new registration framework^2^ which permits both B-spline smoothing on the update (“viscous”) and total (“elastic”) displacement fields at each iteration (cf analogous Gaussian, i.e., Demons, implementation^3^).

Continuing from the work of Christensen et al. (1996) and subsequent exploration into the mathematical formalisms of diffeomorphisms (e.g., Dupuis and Grenander, 1998), the well-known Large Deformation Diffeomorphic Metric Mapping (LDDMM) algorithm was proposed in Beg et al. (2005). In contrast to the mapping produced by Christensen et al. (1996), LDDMM yields the geodesic solution in the space of diffeomorphisms between two images. Since its introduction, LDDMM has inspired much innovation in the image registration literature. Applying the log-Euclidean framework of Arsigny et al. (2006), DARTEL (Diffeomorphic Anatomic Registration using Exponential Lie algebra) uses a constant velocity field parameterization to provide a fast, diffeomorphic alternative (Ashburner, 2007). Additionally, symmetrical considerations in the velocity field parameterization are discussed in Avants et al. (2008) in the context of a cross correlation similarity metric. By explicitly symmetrizing the LDDMM formulation, this Symmetric Normalization (SyN) approach minimizes the bias of the resulting transformation when selecting the “fixed” and “moving” images. A greedy version of this algorithm has proven successful in neuroimaging (Klein et al., 2009) and pulmonary (Murphy et al., 2011) applications as well as in multi-atlas label fusion (Wang et al., 2013).

Although many extensions of LDDMM rely on some form of Gaussian convolution for regularization (e.g., Risser et al., 2011), there has been significant interest in constraining FFD approaches to the space of diffeomorphisms. An early attempt reported in Rueckert et al. (2006) enforced diffeomorphic transforms by concatenating multiple FFD transforms, each of which is constrained to describe a one-to-one mapping. Modat et al. (2011) incorporated the log-Euclidean framework for enforcing diffeomorphic transformations and ensuring invertibility. Similarly, the work of De Craene et al. (2011) provided a full LDDMM-style algorithm based on B-splines called *temporal FFD* in which the time-varying velocity field is modeled using a 4-D B-spline object (3-D + time). Numerical Eulerian integration of the mapping propagated within the velocity field yields the transform between parameterized time points.

As alluded to earlier, B-spline approximation can also be used for regularizing time-varying vector fields in an analogous fashion as Gaussian convolution. In this vein, and similar to De Craene et al. (2011), we reported in Tustison and Avants (2012a,b) the use of an *n*-D + time B-spline object to represent the characteristic velocity fields. However, we use the DMFFD formulation to improve the solution convergence. This also facilitates modeling temporal periodicity and the enforcement of stationary boundaries. Both this work and our earlier work (Tustison et al., 2009) demonstrate that the DMFFD framework is potentially applicable to the entire gamut of diffeomorphic registration algorithms and provides alternative smoothing possibilities with different continuity properties (e.g., *C*^2^ vs. *C*^3^).

The two regularization approaches (i.e., Gaussian convolution vs. DMFFD), however, produce characteristically different solutions. In addition to smoothing kernel differences, Gaussian convolution tends to “flatten” the signal in contrast to an approximation or fitting of the signal provided by the DMFFD approach. Also, whereas Gaussian convolution operates entirely within discretized space, the B-spline approximation routine constructs a continuous object prior to any voxelwise reconstruction of the sampled fields. Interestingly, a similar comparison was made with respect to Gaussian derivative estimation (Bouma et al., 2007). Although typically estimated using truncated, discrete Gaussian convolution, an alternative based on B-spline approximation demonstrated superior performance with similar computational cost.

Three popular diffeomorphic algorithms and their DMFFD analogs (LDDMM, DARTEL, and SyN) were implemented by the authors as part of the recent refactoring of the open source Insight Toolkit (ITK) although related work had been previously implemented within the popular Advanced Normalization Tools (ANTs)^4^. Given the popularity and excellent performance of the greedy variant of SyN, our evaluation focus in this work is its B-spline analog which we denote as “B-spline SyN” or “DMFFD SyN.” Evaluations of the respective algorithmic instantiations are performed using the antsRegistration program found in the ANTs repository (also originally developed by the authors). This permits a direct algorithmic comparison as potential sources for implementation bias have been reduced (Tustison et al., 2013). Additionally, in the spirit of open science, all text, figures, and scripts to reproduce the results contained in this work are publicly available online^5^.

## 2. Material and methods

### 2.1. Theoretical overview

Given the spatial domain Ω of *d*−dimensionality defined over image *I*, a diffeomorphic mapping, ϕ, parameterized over *t* ∈ [0, 1] transforms the image *I* to the target image *J* using *I* ◦ ϕ(**x**, 1) where the geodesic path ϕ(**x**, *t*) is described by (Beg et al., 2005)

(1) $$\underset{\phi }{\inf}\left(\int_{0}^{1}\|v(t){\|}_{L}^{2}dt+\int_{\Omega }|I◦\phi^{-1}(x,1)-J{|}^{2}d\Omega \right).$$

ϕ is generated as the solution of the ordinary differential equation

(2) $$\frac{d\phi (x,t)}{dt}=v(\phi (x,t),t),\phi (x,0)=Id$$

where *v* is a time-dependent smooth field (as dictated by the functional norm *L*), *v*: Ω × *t* → R^*d*^. Diffeomorphic mappings between parameterized time points {*t*_*a*_, *t*_*b*_} ∈ [0, 1] are obtained from Equation (2) through integration of the transport equation, viz.

(3) $$\phi (x,t_{b})=\phi (x,t_{a})+\int_{t_{a}}^{t_{b}}v(\phi (x),t)dt.$$

However, as pointed out in Avants et al. (2008), implementations of this standard LDDMM formulation are negatively affected by the lack of optimization symmetry where arbitrary assignment of fixed and moving images could lead to different solutions despite the fact that the theoretical geodesic solution describes the same parameterized path forwards and backwards. This observation led to the symmetric formulation of Equation (1) found in Avants et al. (2008):

(4) $$\begin{array}{l} \underset{\phi_{1}}{\inf}\underset{\phi_{2}}{\inf}(\int_{0}^{0.5}\left(\|v_{1}(t){\|}_{L}^{2}+\|v_{2}(t){\|}_{L}^{2}\right)dt\\ \;+\int_{\Omega }|I◦\phi_{1}^{-1}(x,0.5)-J◦\phi_{2}^{-1}(x,0.5){|}^{2}d\Omega ) \end{array}$$

where

(5) $$\frac{d\phi_{i}(x,t)}{dt}=v_{i}(\phi_{i}(x,t),t),\phi_{i}(x,0)=Id,i\in \{1,2\}.$$

**Figure 1:**
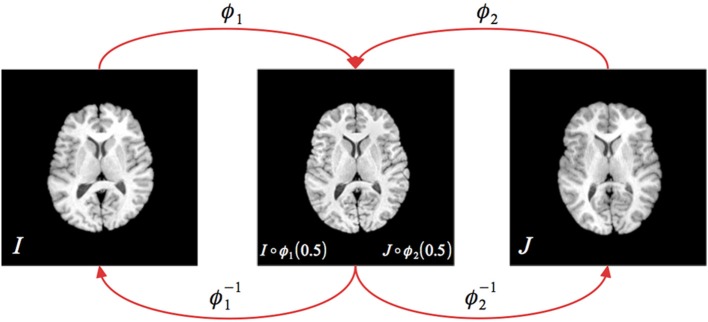
**Illustration of the greedy SyN formulation**. Given images *I*_*A*_ and *I*_*B*_, the symmetric set-up requires finding the two transform pairs (ϕ_1_, ϕ^−1^_1_) (ϕ_2_, ϕ^−1^_2_) which map to/from the respective images to the midway point. During optimization, the update field at each iteration is determined from the metric field gradient taken at the midway point, i.e., ∇ Π_~_ (*I* ◦ ϕ_1_(0.5), *J* ◦ ϕ_2_(0.5)). The full forward and inverse transforms are found through composition, i.e., ϕ = ϕ_1_ ◦ ϕ^−1^_2_ and ϕ^−1^ = ϕ_2_ ◦ ϕ^−1^_1_.

With extension to arbitrary similarity metric choice, the second term is replaced with

(6) $$\int_{\Omega }\Pi_{\sim }\left(I◦\phi_{1}^{-1}(x,0.5),J◦\phi_{2}^{-1}(x,0.5)\right)d\Omega$$

with a popular choice for Π_~_ being a local neighborhood cross correlation (Avants et al., 2008, 2011). Note that *t* is parameterized in opposite directions between ϕ_1_ and ϕ_2_. A diagrammatic illustration of the explicit symmetry associated with SyN is shown in Figure 1.

#### 2.1.1. St. nava's theory of greed and original SyN

Although presenting a rigorous framework for image registration solutions with desirable properties, the complexity of these diffeomorphic methodologies requires substantial computational resources. For typical 3-D neuroimaging applications, the corresponding solutions require numerical integration over and storage of 4-D velocity fields at each iteration which is limiting for many common computational platforms.

Therefore, in addition to the full-scale SyN offering described in Avants et al. (2008), the authors therein provided a “greedy” alternative which has demonstrated superior performance in different applications (Klein et al., 2009; Avants et al., 2011; Murphy et al., 2011)^6^ while simultaneously being capable of running with limited computational resources. This is due to the restriction of the discrete, time-parameterized velocity field samples to their respective endpoints, i.e., the *v*_*i*_(**x**, *t*) are sampled at *t* ∈ {0, 0.5, 1} implying simultaneous storage of only four transform vector fields ϕ_1_(**x**), ϕ^−1^_1_(**x**), ϕ_2_(**x**), and ϕ^−1^_2_(**x**) (cf Figure 1). Furthermore, the forward and inverse mappings are guaranteed to be consistent within the discrete domain i.e., ∥ϕ^−1^_*i*_(ϕ_*i*_) − **Id**∥^2^ < ϵ. Since the greedy SyN framework is the focus of the evaluation, the algorithmic steps are briefly sketched in Algorithm 1.

**Algorithm 1:** **Greedy SyN algorithm**.

ϕ_*i*_ ← **Id**, ϕ^−1^_*i*_ ← **Id** ⊳ *i* ∈ {1, 2}
**for all** image resolution levels **do**
*n* ← 1
**While** not converged **do**
$v_{1}^{n}\leftarrow \nabla \Pi_{\sim }\left(I◦\phi_{1}^{n-1},J◦\phi_{2}^{n-1}\right)$
$v_{2}^{n}\leftarrow \nabla \Pi_{\sim }\left(J◦\phi_{2}^{n-1},I◦\phi_{1}^{n-1}\right)$
*v*^*n*^_*i*_ ← *S*_*v*_(*v*^*n*^_*i*_) ⊳ *S*_*v*_ is a smoothing operation on the update transform field
ϕ^*n*^_*i*_ ← *S*_ϕ_(*v*^*n*^_*i*_ ◦ ϕ^*n*−1^_*i*_) ⊳ *S*_ϕ_ is a smoothing operation on the total transform field
${\left(\phi_{i}^{n}\right)}^{-1}\leftarrow Inv\left(\phi_{i}^{n},{\left(\phi_{i}^{n-1}\right)}^{-1}\right)$ ⊳ Inverse field estimation described in Avants et al. (2008)
*n* ← *n* + 1
**end while**
upsample current ϕ_*i*_ and ϕ^−1^_*i*_ to next resolution level ⊳ *i* ∈ {1, 2}
**end for**
**return** ϕ ← ϕ_1_ ◦ ϕ^−1^_2_, ϕ^−1^ ← ϕ_2_ ◦ ϕ^−1^_1_

#### 2.1.2. Directly manipulated free-form deformation diffeomorphic analogs

Although several velocity field regularization operators have been proposed, many algorithmic instantiations default to Gaussian smoothing due to its simplicity both in implementation and complexity terms. A viable and practical alternative is the DMFFD approach based on B-splines for explicit regularization of vector fields.

Given the similarity metric Π_~_, the *d*-dimensional update field (i.e., preconditioned gradient field), δ*v*_*i*_1_, …, *i*_*d*__, is given by

(7) $$\delta v_{i_{1},\ldots ,i_{d}}=\frac{\sum_{c=1}^{N_{\Omega }}{\left(\frac{\partial \Pi_{\sim }}{\partial x}\right)}_{c}\prod_{j=1}^{d}B_{i_{j}}(x_{j}^{c})\cdot \frac{\prod_{j=1}^{d}B_{i_{j}}^{2}(x_{j}^{c})}{\sum_{k_{1}=1}^{r+1}\ldots \sum_{k_{d}=1}^{r+1}\prod_{j=1}^{d}B_{k_{j}}^{2}(x_{j}^{c})}}{\sum_{c=1}^{N_{\Omega }}\prod_{j=1}^{d}B_{i_{j}}^{2}(x_{j}^{c})}$$

where the set of *B*(·) are the univariate B-spline basis functions for separately modulating regularity in the solution for each parametric dimension, *N*_Ω_ is the number of voxels in the reference image domain, *r* is the spline order in all dimensions,^7^ and $\frac{\partial \Pi_{\sim }}{\partial x}$ is the spatial similarity metric gradient at voxel *c*^8^.

Similarly, in the case of *d*-dimensional time-parameterized diffeomorphic image registration, the time-dependent velocity field can be represented as a (*d* + 1)-dimensional B-spline object

(9) $$v(x,\;t)=\sum_{i_{1}=1}^{X_{1}}\;\ldots \;\sum_{i_{d}=1}^{X_{d}}\sum_{i_{t}=1}^{T}v_{i_{1},\;\ldots ,\;i_{d},\;i_{t}}B_{i_{t}}(t)\prod_{j=1}^{d}B_{i_{j}}(x_{j})$$

where *v*_*i*_1_, …, *i*_*d*_, *i*_*t*__ is a (*d* + 1)-dimensional control point lattice characterizing the velocity field. The preconditioned gradient analog of Equation (7) for updating the time-varying velocity field control point lattice is

(10) $$\delta v_{i_{1},\;\ldots ,\;i_{d},\;i_{t}}=(\sum_{c=1}^{N_{\Omega }\times N_{t}}{\left(\frac{\partial \Pi_{\sim }}{\partial x}\right)}_{c}B_{i_{t}}(t^{c})\prod_{j=1}^{d}B_{i_{j}}(x_{j}^{c})\;\cdot \;\frac{B_{i_{t}}^{2}(t^{c})\prod_{j=1}^{d}B_{i_{j}}^{2}(x_{j}^{c})}{\sum_{k_{1}=1}^{r+1}\ldots \sum_{k_{d}=1}^{r+1}\sum_{k_{t}=1}^{r+1}B_{k_{t}}^{2}(t^{c})\prod_{j=1}^{d}B_{k_{j}}^{2}(x_{j}^{c})})\;\cdot \;{\left(\sum_{c=1}^{N_{\Omega }\times N_{t}}B_{i_{t}}^{2}(t^{c})\prod_{j=1}^{d}B_{i_{j}}^{2}(x_{j}^{c})\right)}^{-1}$$

which takes into account the temporal locations of the dense gradient field sampled in *t* ∈ [0, 1]. *N*_*t*_ and *N*_Ω_ are the number of time point samples and the number of voxels in the reference image domain, respectively^9^.

For regularization of constant velocity fields, e.g., SyN or DARTEL, updating the field is performed using Equation (7). In the case B-spline SyN, this applies to the smoothing operators *S*_*v*_ and *S*_ϕ_ in **Algorithm 1** although best performance (at least for the data described in this work) typically employs no smoothing on the total transform field, i.e., *S*_ϕ_ is such that *S*_ϕ_(ϕ^*n*^_*i*_) = ϕ^*n*^_*i*_.

### 2.2. Implementation

Three diffeomorphic algorithms described previously utilizing Gaussian convolution smoothing and their DMFFD counterparts are available through the following classes in the ITK repository:

- **LDDMM:**
  - itk::TimeVaryingVelocityFieldTransform
  - itk::TimeVaryingVelocityFieldTransform
    ParametersAdaptor
  - itk::TimeVaryingVelocityFieldImage
    RegistrationMethodv4
  - itk::GaussianSmoothingOnUpdateTime
    VaryingVelocityFieldTransform
  - itk::TimeVaryingBSplineVelocityField
    Transform
  - itk::TimeVaryingBSplineVelocityField
    ImageRegistrationMethodv4
  - itk::TimeVaryingBSplineVelocityField
    TransformParametersAdaptor
- **DARTEL:**
  - itk::ConstantVelocityFieldTransform
  - itk::ConstantVelocityFieldTransform
    ParametersAdaptor
  - itk::GaussianExponentialDiffeomorphic
    Transform
  - itk::GaussianExponentialDiffeomorphic
    TransformParametersAdaptor
  - itk::BSplineExponentialDiffeomorphic
    Transform
  - itk::BSplineExponentialDiffeomorphic
    TransformParametersAdaptor
- **SyN:**
  - itk::SyNImageRegistrationMethod
  - itk::BSplineSyNImageRegistrationMethod

These and other classes (e.g., similarity metrics, optimization methods, and utility classes) were developed as part of the ITKv4 registration framework refactoring. Much of the original ITK image registration infrastructure was left intact including the so-called “sparse” transforms such as various rigid (versor, Euclidean) and other linear transforms. The transform classes contributed by our group were, including those listed above, were meant to augment what already existed. All the transforms listed above are derived from the itk::DisplacementFieldTransform class which permits specification of a transform described by a sampled displacement field. The derived classes are then modified according to the different transform constraints. Other types of classes are used to coordinate the image registration process. The itk::ImageRegistrationMethodv4 is the base interface for performing all image registration steps. Smoothing and resampling for multi-resolution image registration is performed in this class as is the calling of the selected optimizer. Output consists of a single optimized transform. Multiple instantiations of this class in series, in conjunction with the itk::CompositeTransform class, are used to optimize a composition of transforms. Some of the diffeomorphic approaches do not easily fit into this generalized optimization framework necessitating specialized method classes such as those listed above. The adaptors^10^ are used to modify the parameters between resolution levels during the course of transform optimization within the methods classes. For example, the resolution of the displacement field transforms follows that of the image resolution and the updating is handled by the corresponding transform adaptors. Further details can be found in the documentation provided within the classes themselves^11^.

The class itk::BSplineScatteredDataPointSet ToImageFilter underlies all DMFFD regularization which is an implementation of the methods described in Tustison and Gee (2006). Although applicable to various scenarios (e.g., curve and surface estimation), it has been optimized for imaging applications and multi-threaded for fast processing on suitable machines. Additionally, numerical integration for solving Equations (2) and (3) utilizes Runge-Kutta which provides a more stable alternative than other methods (Press et al., 2007). Implementation is provided in the class itk::TimeVaryingVelocityFieldIntegration ImageFilter.

A complete packaging of these classes has been made available as part of our ANTs toolkit^12^. The antsRegistration program^13^ takes advantage of the enhanced ITKv4 registration framework and was developed by the authors to provide a robust and versatile solution for a wide variety of image registration applications. The basic conceptualization for use is that one can set-up any number of registration “stages” with each stage being characterized by a specified transform. For example, a representative command call is as follows:

**Listing 1:**
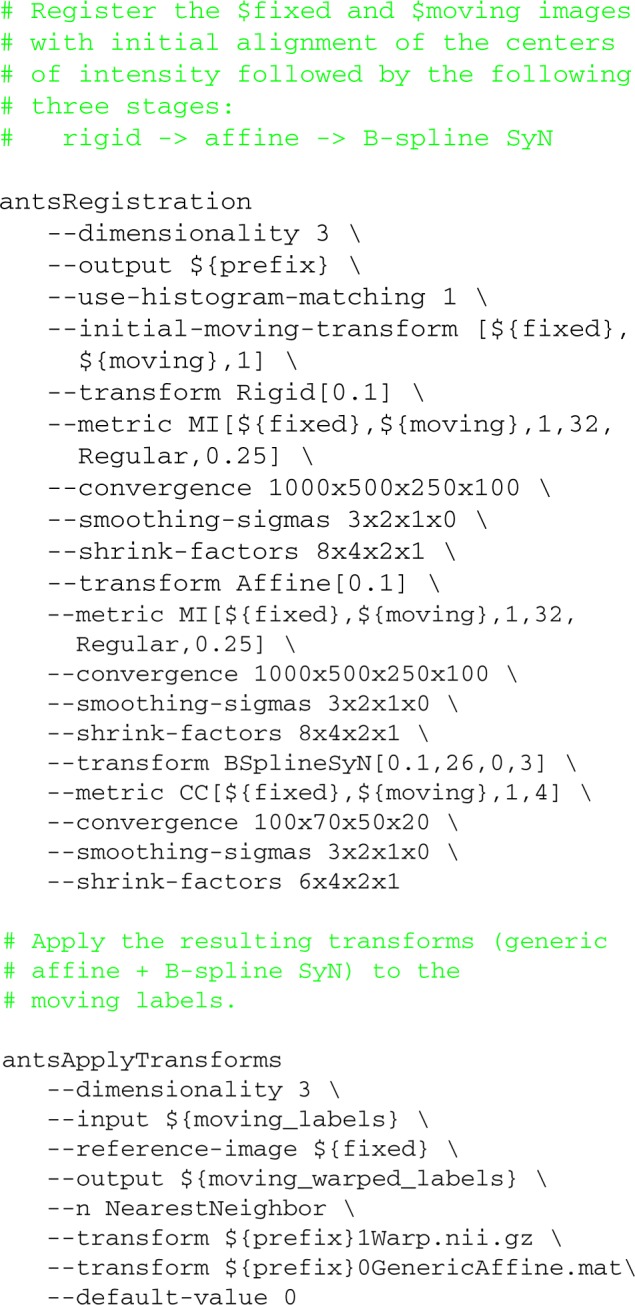
**Representative script containing antsRegistration and antsApplyTransforms command calls used for evaluation**.

In this example, we first calculate an initial translation transform by aligning the centers of (intensity) mass (although alignment based on other features is possible)^14^. The resulting transform is then used as input for determining an optimal rigid transform. Serial propagation of the resulting composite transform continues until all optimal transforms have been determined. Optimization for each stage is determined by the specified general parameters including: smoothing and downsampling of fixed and moving images, convergence criteria (including number of iterations per resolution level) and metric (or metrics). Any pair of images can be specified per metric per stage^15^.

Although the resulting transforms for each stage can be written to disk as output, the default output consists of a condensed set of transforms where compatible transforms have been composed to a single transform. For example, in the above command call, the initial translation, rigid, and affine transforms are combined into a single generic affine transform file with the results of the deformable transform consisting of discrete vector fields. The output transform files can then be applied using the antsApplyTransforms program which permits composition of any number of transform files with different interpolation schemes. For both programs interpolation is never performed more than once.

### 2.3. Evaluation data

In the well-known Klein comparative study (Klein et al., 2009), 14 image registration algorithms were evaluated based on performance on publicly available labeled brain data. For our evaluation, we used these same data. Specifically, we used the data sets denoted as:

- CUMC12
- IBSR18
- LPBA40
- MGH10

which are available for download from Arno Klein's website^16^.

The number of subjects per cohort is provided in the denotation. Table 1 summarizes core information about the data sets used. Further details of these first four labeled brain data (e.g., labeling protocol, data sources) are given in Klein et al. (2009). We also include the labeled brain data provided at the MICCAI 2012 Grand Challenge and Workshop on Multi-Atlas Labeling^17^ which we denote as MAL35. This T1-weighted MRI data set consists of 35 subject MRIs taken from the Oasis database^18^. The corresponding labels were provided by Neuromorphometrics, Inc^19^. under academic subscription.

**Table 1:** **Brief overview of the data sets used for the SyN vs. B-spline SyN comparison**.

**Cohort**	**Resolution**	**Number of labels**	**Preprocessing**
CUMC12	[0.86, 0.86, 1.5]	131	Rotated to “cardinal” pose
IBSR18	[1, 1.5, 1]	96	Aligned to talaraich, bias corr.
LPBA40	[0.86, 1.5, 0.86]	56	Registered to MNI305, bias corr.
MGH10	[1, 1, 1]	106	Affine-registered to MNI152, bias corr.
MAL35	[1, 1, 1]	138	Average of 3–4 acquisitions, bias corr.

Comparative evaluation of the two SyN registration approaches was performed within each cohort using a “pseudo-geodesic” approach. Instead of registering every subject to every other subject within a data set, we generated the transforms from each subject to a cohort-specific shape/intensity template. Not only does this reduce the computational time required for finding the pairwise transforms between subjects but prior work has demonstrated improvement in registration with this approach over direct pairwise registration (Klein et al., 2010a). Since the two algorithms have been implemented within the same framework, all registration parameters are identical (i.e., linear registration stage parameters, winsorizing values, etc.) except for the parameters governing the smoothing of the gradient field.

**Figure 2:**
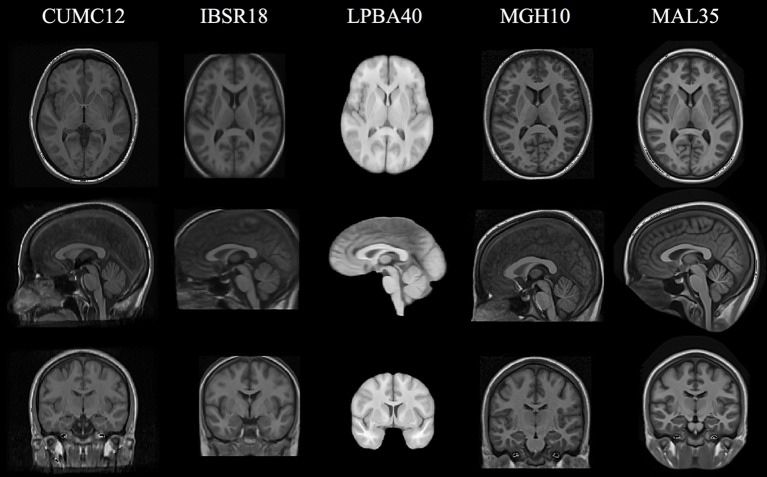
**Canonical views for each of the five cohort-specific templates generated using the ANTs tools as described in Avants et al. (2010)**. The pseudo-geodesic transform between subjects is created from the composition of transforms to/from the relevant template.

**Figure 3:**
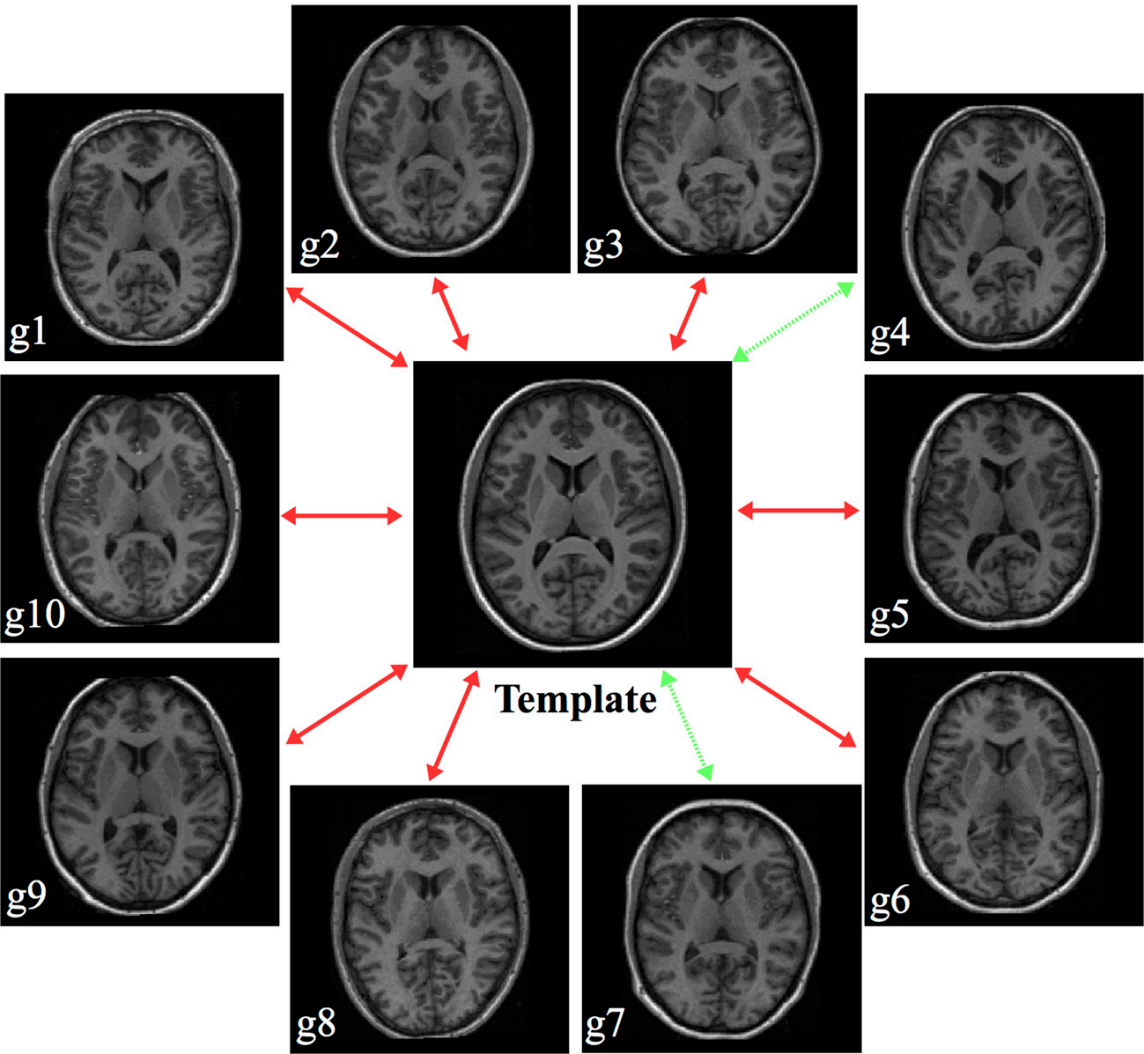
**Illustration of generating a pseudo-geodesic for any two subjects within the MGH10 cohort**. Once the transforms between the template and each subject are calculated, the mapping between any two subjects is found by composition of forward and inverse transforms. For example, in the MGH data set, the pseudo-geodesic transform to map Subject g4 to Subject g7 is found by composing the forward transform from g4 to the template with the inverse transform from the template to g7 (green dashed lines).

The cohort templates were built using the ANTs script ants MultivariateTemplateConstruction.sh which is a multivariate implementation of the work described in Avants et al. (2010). Canonical views for each of the five templates used for this study are given in Figure 2. Since calculation of the transform from each subject to the template also includes generation of the corresponding inverse transform, the total transformation from a given subject to any other is determined from the composition of transforms mapping through the template. An example illustration of the geodesic approach is given in Figure 3.

**Figure 4:**
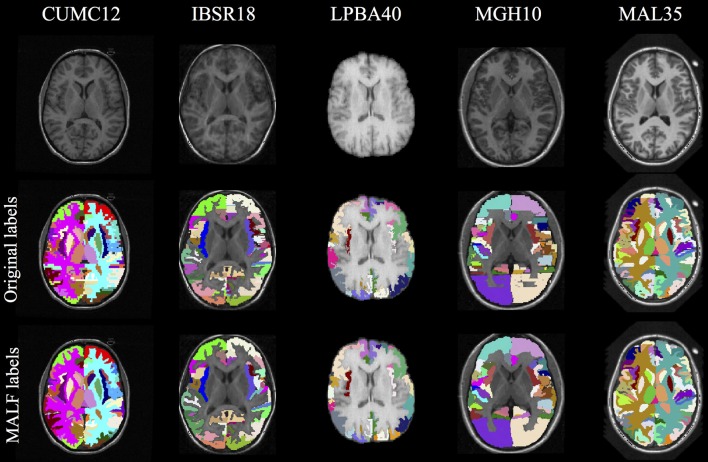
**Axial views of sample labelings for a member of each data set**. The second row consists of the original labelings with the third row being refined versions of those labelings using the MALF algorithm (Wang et al., 2013). These refinements provide more consistency between labelings and improved comparative assessments between algorithms.

Additionally, we refined the labelings for each subject of each cohort using the multi-atlas label fusion algorithm (MALF) developed by Wang et al. (2013) which is also distributed with ANTs. For a given subject within a data set, every other subject was mapped to that subject using the pseudo-geodesic transform. The set of transformed labelings were then used to determine a consensus labeling for that subject. This was to minimize the obvious *observer dimensionality artifacts* where manual raters observe and label in a single dimension at a time. This is most easily seen in the axial or sagittal views of the different cohorts as labelings were done primarily in the coronal view (see Figure 4). We include both sets of results. This provides two sets of labels per subject for evaluation^20^.

## 3. Results

As mentioned previously, a template was constructed for each data set (cf Figure 2) from all cohort images. Subsequently, each image was registered to its corresponding template using either SyN or B-spline SyN as described previously (prior linear registration stages were identical between the two algorithms). As a brute-force parameter exploration is not a part of this work, we rely on previously reported research (Klein et al., 2009; Avants et al., 2011) and our own experience as authors/developers of the algorithm/software to select parameters which demonstrate robust performance across data sets. For both algorithms, the gradient step was 0.1 for each of the four multi-resolution levels with shrink factors of {6, 4, 2, 1} and Gaussian smoothing for each of those levels being 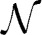
(0, {9, 4, 1, 0}) in terms of voxels. The number of iterations per level were {100, 100, 70, 20} with a convergence threshold of 10^−9^ and window size of 15 iterations^21^.

All processing was performed using the linux cluster at the University of Virginia^22^ using the PBS Pro queuing system for managing resources. The perl scripts used to create the jobs for the cluster are included in the github account associated with this evaluation. For the data sets used in this study, times for B-spline SyN were approximately 15–40% greater than Gaussian-based SyN using single-threading and a dense metric gradient sampling. Timing data for specific data sets are given in Table 2 Timing includes both rigid and affine transform optimizations.

**Table 2:** **Timing (in hours) per registration for both SyN algorithms across data sets**.

**Cohort**	**SyN**	**B-spline SyN**
CUMC12	8.6 ± 2.3	9.6 ± 2.1
IBSR18	8.3 ± 2.7	10.7 ± 2.8
LPBA40	6.6 ± 0.7	9.5 ± 1.4
MGH10	4.7 ± 1.5	5.8 ± 1.2
MAL35	10.6 ± 1.9	14.2 ± 2.0

The only difference between the two registration settings consists of the Gaussian and B-spline parameters governing the update field smoothing, *S*_*v*_. In our experience, smoothing of the total field did not improve the results, at least for these data (which conforms with our experience with other data), so the total field smoothing, *S*_ϕ_ is 0 for both registration approaches. Specifically, the chosen parameters for the SyN algorithm were: *S*_ϕ_ = 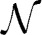
(0, 0) and *S*_*v*_ = 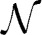
(0, 3) in voxel terms^23^. Although our experience with B-spline SyN is much more limited, we were able to choose comparable parameters based on a knot spacing for the update field of 26 mm at the base level which is reduced by a factor of two for each succeeding multiresolution level. This yields a final knot spacing of 3.25 mm^24^. For comparison, after selecting these smoothing parameters we discovered in the supplementary material of Klein et al. (2009) the similarity to the gradient smoothing parameter for the IRTK FFD algorithm which also used four multi-resolution levels with an initial knot spacing of 20 mm per dimension for a final knot spacing of 2.5 mm.

**Figure 5:**
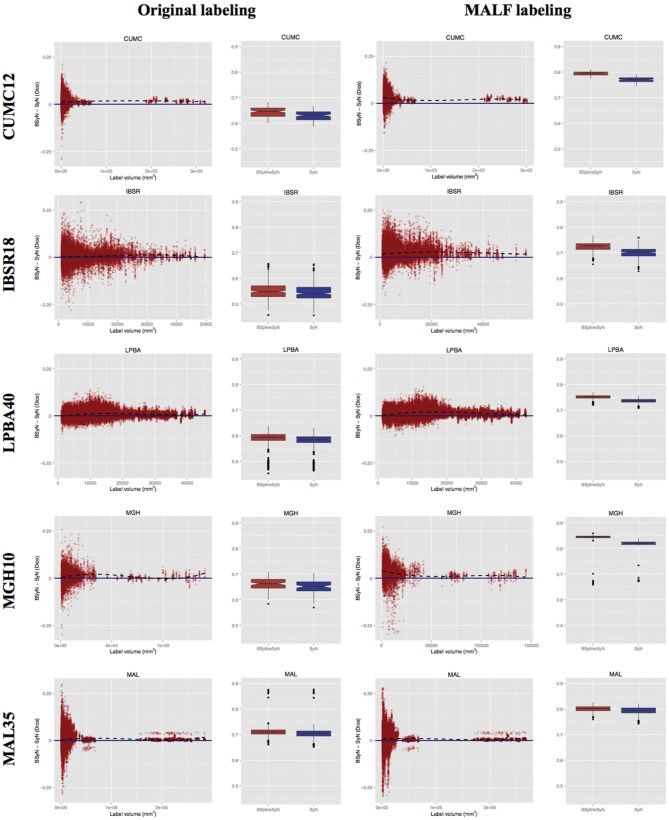
**Dice results for both algorithms for each subject warped to every other subject using the pseudo- geodesic transform**. Each row corresponds to one of the five data sets used for evaluation. For each data set we include a plotting of all individual label Dice results by volume and a combined label box plotting. The **left** and **right** halves show the respective results for original and MALF-derived labelings. The black dashed regression line (*y* ~ *x*^3^) illustrates how performance difference varies with label volume for each cohort.

Quality of overlap using the Dice similarity metric was determined from the transformed labels using the open source ITK implementation described in Tustison and Gee (2009). Both the original labels and MALF labels were warped to the fixed image for comparison using nearest neighbor interpolation. A joint Dice metric value was calculated from the combined labels for each cohort for each of the two SyN methods. These values are rendered in notched box plot format in Figure 5. Non-overlapping notches indicate approximately statistically significantly different median values at the 95% confidence level (McGill et al., 1978). For all data sets, the B-spline SyN variant showed a small but statistically significant improvement in overall Dice values. In order to provide a more complete picture of performance differences, we also accounted for label volumetric considerations (Rohlfing, 2012). In Figure 5 we plotted the Dice value difference between each SyN variant (B-spline SyN—SyN) for each label of each intra-subject registration pair within a data set vs. the volume of the label in the fixed image. Values above and below the dashed line indicate better regional performance for B-spline SyN and SyN, respectively.

**Figure 6:**
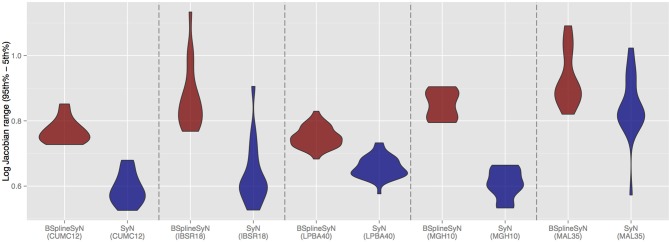
**Violin plots of the range of log Jacobian values (95th%—5th%) for all deformable transforms from each subject to its corresponding template**. B-spline SyN demonstrates a tendency to produce a much greater range of log Jacobian values.

**Figure 7:**
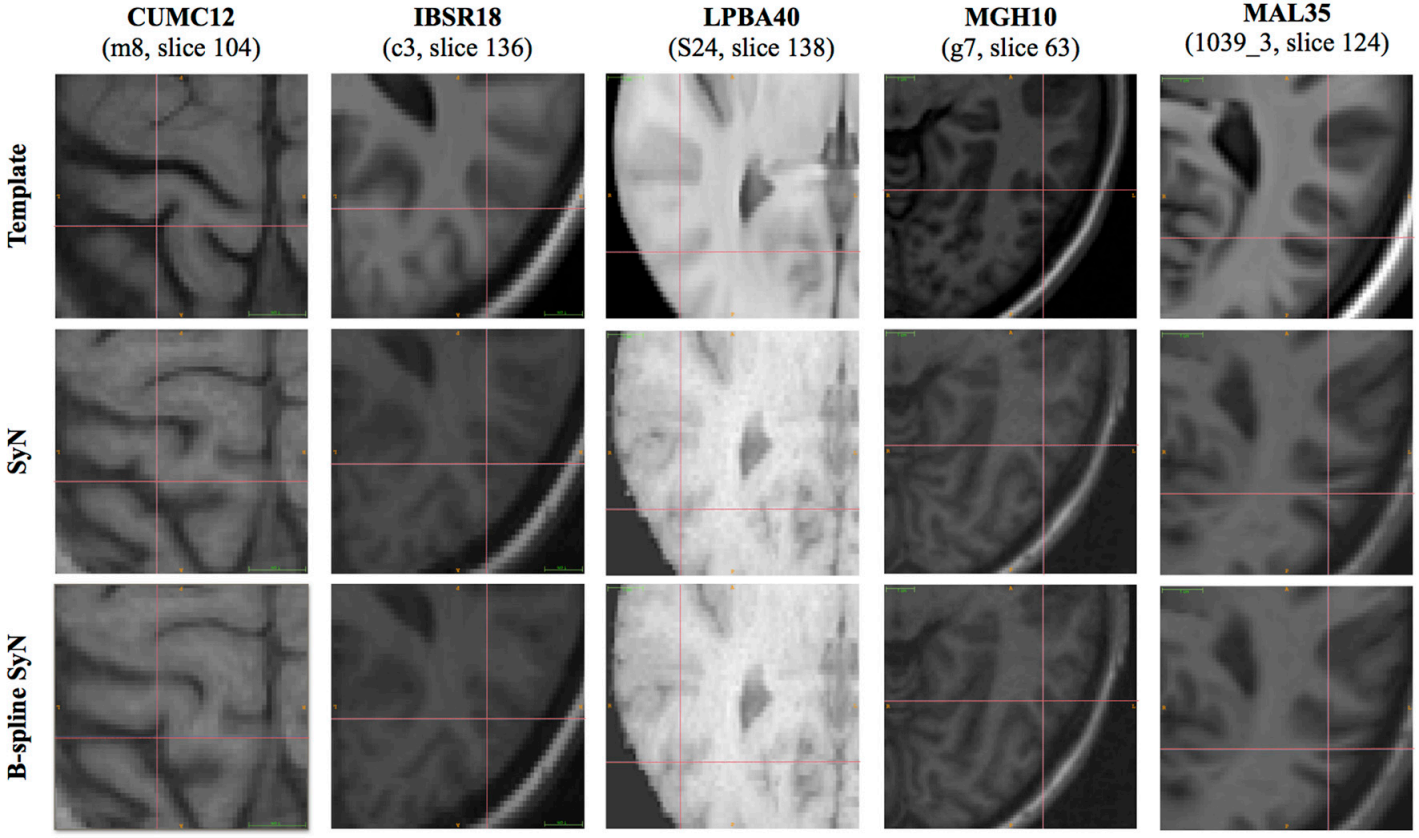
**Randomly selected axial slices showing qualitative differences between SyN and B-spline SyN**. Crosshairs indicate regions of maximal Jacobian difference.

To further characterize the deformable transform differences, we calculated the log of the Jacobian determinant of the transformations from each subject to the template and tabulated statistical information within the brain region only. A noticeable difference between the two algorithms was the respective range of values in log Jacobians. We plotted the (95th%—5th%) for each algorithm across all data sets in Figure 6. It is apparent that B-spline SyN results exhibit a much greater range of deformation. Qualitative differences are shown in Figure 7. In order to ensure randomness to minimize presentation bias in illustrating qualitative results (and given the relative poor performance in humans as random number generators Wagenaar, 1972), we used R to generate uniform random numbers for both subject and axial slice selection. We then used the log Jacobian images to locate regions of maximal difference between the SyN and B-spline SyN results. From Figure 7 it is quite apparent that the results are very similar which is to be expected considering the almost identical algorithmic make-up between the two approaches (e.g., similarity metric, implementation, linear transforms). However, there are subtle differences particularly in the cortex which help explain both the relative difference in Jacobian and Dice distribution.

## 4. Discussion and conclusions

B-spline SyN produced slightly greater Dice values than the original SyN. Although actual differences are relatively small, they are statistically significant. By implementing both algorithms in the same code base, we are not only able to eliminate non-regularization components of the registration but we are also able to eliminate implementation differences. Thus, performance disparity can be isolated to smoothing choice. However, even with this restricted focus there are various reasons for the evaluation outcome. These include the approximation-vs.-convolution distinction mentioned earlier for the two regularization approaches (which could also explain the reason why the range of log Jacobian values tend to be significantly higher for B-spline SyN). Also, the fact that the regularization for B-spline SyN is theoretically continuous whereas the truncated Gaussian convolution is only a discrete approximation could be a potential factor.

Additional observations of interest concern the differences in results between the MALF and original labelings for all data sets. Not only was there an overall increase in performance for both algorithms with the MALF labels for all cohorts, but there was also an increase in performance disparity relative to the variance in the resulting Dice values. A possible explanation for this, and one that seems quite plausible, is that the MALF labelings are derived from registering a cohort to the target subject and then performing a consensus labeling (Wang et al., 2013). Both these steps are heavily reliant on image intensity information (in fact, both use a form of correlation as the similarity measure for optimization). Since the MALF “correction” tends to group labeled regions according to the same metric used for establishing anatomical correspondences, alignment of these labeled regions seems much more likely which would result in higher Dice metrics. A related effect for labeled data in general is being currently investigated by the authors. From the label volume vs. Dice difference plots, there is no immediately discernible pattern of performance variation with region size. However, a possible confound is that region definitions vary between cohorts. Although we did not look at region vs. performance difference variation within cohorts, such inquiry is certainly possible as we have made the resulting csv files available with the github repository associated with this work.

Relative to Klein's study (Klein et al., 2009), it should be noted that the SyN implementation used in that study is found in the ANTS program which is a precursor of the antsRegistration program described earlier. Although the theoretical aspects are the same, there are substantial implementation differences between the two programs. In addition, several parameters varied between the two studies which translated into a more aggressive metric and gradient step in addition to fewer levels and iterations^25^. In this study we took a more conservative approach based on our continued development and experience resulting in parameters which have proven useful in our cortical thickness pipeline (encapsulated in the ANTs script antsCorticalThickness.sh) and our experience with the MICCAI 2013 SATA challenge data. We also ran our own internal experiments with Gaussian-based SyN (both ANTS and antsRegistration) using the same conservative parameters on the MAL35 data for which the latter demonstrated slightly improved performance over the former.

Despite the thorough evaluation with multiple data sets, we readily acknowledge the limitations of this study including a very focused application, i.e., healthy brains of a single modality, and absence of a thorough exploration of parameter selection and sensitivity. Although such work might be beneficial (e.g., by aiding other researchers in parameter selection), characterizing parameter permutations of potential interest would expand the current work far beyond its intended scope. However, in addition to this work, SyN has also been previously evaluated in Klein et al. (2009) and Avants et al. (2011) and, based on additional experience and application, the parameter set was modified each time but still yielded excellent performance providing evidence for flexibility in parameter selection. Outside of a range of parameters based on sound engineering principles, experience, and intimate knowledge of both the corresponding algorithms and software, determination of optimal generic parameters even for a specific application is difficult. In fact, the “No Free Lunch Theorem” Wolpert and Macready (1997) emphasizes the importance of prior knowledge in tuning optimization algorithms for a particular application.

One of the advantages that has not been explored in this work is the use of B-spline SyN for small deformation estimation problems such as in pulmonary or cardiac applications. Such problems typically require greater regularization which implies larger discrete kernels for Gaussian convolution. A related issue concerns applications involving severely anisotropic data where the continuous nature of the DMFFD approach might help over Gaussian convolution. Also, we emphasize that underlying the DMFFD approaches is a fitting routine for sparse and scattered data which offers added flexibility over smoothing using discrete convolution where the latter implies regularly placed data on a rectilinear grid for conventional implementations. This advantage could translate into faster running times if only select points are used to drive the registration or make possible more complex registration scenarios involving data arranged continuously within a finite domain (e.g., Tustison et al., 2011). Finally, the possibility of varying data confidence values, as introduced in Tustison and Gee (2006) with DMFFD-based routines would permit incorporation of spatial preferential weighting (i.e., additional prior knowledge) during optimization. Ongoing work will continue to explore these issues.

A significant amount of research has been devoted to image registration algorithmic development. Given their many salient characteristics particularly with respect to large deformation estimation constrained by topological continuity, diffeomorphic registration approaches have been a particular focus in the neuroimaging community. However, many groups continue to find success with non-diffeomorphic FFD methods (e.g., Rueckert et al., 1999; Klein et al., 2010b). Using our DMFFD framework, B-spline regularization is easily adapted into the diffeomorphic registration framework and performs well compared to analogous algorithms which we demonstrated in this work for the case of the widely-used SyN.

### Conflict of interest statement

The authors declare that the research was conducted in the absence of any commercial or financial relationships that could be construed as a potential conflict of interest.

## Footnotes

^1^This phenomenon is explained in greater detail in Tustison et al. (2009) but, briefly, the distribution of the uniform B-spline basis functions spanning the image domain produces long ravines in the image registration energy topography. These topographical features cause an inefficient back-and-forth traversal in classic steepest descent optimization, also known as “hemstitching.”

^2^http://www.itk.org/Doxygen/html/classitk_1_1BSplineSmoothingOnUpdateDisplacementFieldTransform.html.

^3^http://www.itk.org/Doxygen/html/classitk_1_1GaussianSmoothingOnUpdateDisplacementFieldTransform.html.

^4^http://stnava.github.io/ANTs.

^5^https://github.com/ntustison/BSplineMorphisms.

^6^SyN was also the standard registration for the MICCAI 2013 Workshop on Segmentation: Algorithms Theory, and Applications (SATA) and corresponding challenge (https://masi.vuse.vanderbilt.edu/workshop2013).

^7^In terms of implementation, spline orders can be specified separately for each dimension but, for simplicity, we only specify a single spline order.

^8^For comparison, FFD image registration uses the calculated gradient which, for a single control point is evaluated using (in discretized form assuming no other explicit regularization):

(8) $$\delta v_{i_{1},\;\ldots ,\;i_{d}}=\sum_{c=1}^{N_{\Omega }}{\left(\frac{\partial \Pi_{\sim }}{\partial x}\right)}_{c}\prod_{j=1}^{d}B_{i_{j}}(x_{j}^{c}).$$

Intuitively, this can be understood as a distribution of the spatial similarity metric gradient to the set of control points according to the basis function weighting which, due to the local property of B-splines, is only non-zero in a localized region surrounding each sample point. The problematic issue with standard FFD image registration is that the parametric domain of the peripheral B-spline basis functions extends beyond the image boundaries resulting in a relatively lower weighting contribution effect. As a corrective, Equation (7) normalizes the control point gradient contribution by the actual B-spline weighting overlap with the image domain. Note that this modification is first discussed and derived in Hsu et al. (1992) and Lee et al. (1997) in the context of fitting scattered data for a computer graphics audience which was an extension and improvement over Sederberg's original FFD manipulation computer graphics technique (Sederberg and Parry, 1986). Whereas the latter (i.e., FFD) technique performs geometric deformation of the graphics object embedded in a B-spline object via manipulation of the control points, the former (i.e., DMFFD) technique permits direct manipulation of the actual object with the control point values being updated indirectly. The corresponding fast algorithm for scattered data approximation using DMFFD was proposed in Lee et al. (1997). Based on the needs of one of our colleagues, the first author (N.T.) implemented and generalized the work of Lee et al. (1997) which was reported in Tustison and Gee (2006) and was N.T.'s very first ITK contribution. A couple years later the second author (B.A.) requested an FFD implementation for ANTs which N.T. naively assumed to be equivalent to applying Tustison and Gee (2006) to smoothing the metric gradient (a la Demons). The subsequent realization that this assumption was incorrect and figuring out why the algorithm still worked despite it not being a “correct” FFD implementation resulted in Tustison et al. (2009).

^9^Additionally, in Tustison and Gee (2006) it was shown that one could associate each a sample, ${\left(\frac{\partial \Pi }{\partial x}\right)}_{c}$ with a confidence weighting. Thus, in order to enforce stationary boundaries for all described DMFFD-based diffeomorphic algorithms, we assign to the image boundary metric gradients the null vector with a corresponding large confidence value.

^10^We gratefully acknowledge Marius Staring's contribution in providing the broad design of the adaptors.

^11^http://www.itk.org/Doxygen/html/classes.html.

^12^http://stnava.github.io/ANTs/.

^13^The long and short command line help menus can be invoked by the commands ‘antsRegistration -help’ and ‘antsRegistration -h’, respectively.

^14^This step corresponds to the –initial-moving-transform option. Generally, the user can specify an ITK transform for initialization or can perform an initial translation based on either the geometric center of the images, the center of the image intensities, or the origin of the images. See the help menu ‘antsRegistration -help’ for more details.

^15^To help the reader who wishes to explore antsRegistration with specific use of the parameters used for this study and specified above, we have created a script with a simplified interface and placed it in the Scripts/ subdirectory of the ANTs repository. This script, called antsRegistrationSyN.sh takes a fixed and moving image and performs a comprehensive (i.e., rigid → affine → deformable) registration using SyN (‘-t s’) or B-spline SyN (‘-t b’).

^16^http://mindboggle.info/papers/evaluation_NeuroImage2009.php.

^17^https://masi.vuse.vanderbilt.edu/workshop2012.

^18^http://www.oasis-brains.org.

^19^http://Neuromorphometrics.com/.

^20^The MALF labels are available at http://figshare.com/account/projects/196.

^21^As part of the ITKv4 refactoring, we developed several convergence monitoring classes (cf the base class itk::Convergence MonitoringFunction). Instead of testing for convergence between two successive time points, we use a windowed monitoring function which keeps track of a sequence, or window, of energy values and determines the convergence from the slope of a line fitted to the series.

^22^http://www.uvacse.virginia.edu.

^23^Or, in equivalent antsRegistration command line parlance, -t SyN[0.1, 3, 0].

^24^Or, equivalently, -t BSplineSyN[0.1, 26, 0].

^25^Specifically, as provided in the supplementary material of Klein et al. (2009), the command line parameters were: ANTS 3 -m PR[${fixed},${moving},1,2] -o ${output} -r Gauss[2,0] -t SyN[0.5] -i 30x99x11 -use-Histogram-Matching.
